# Analysis of histone modifications at human ribosomal DNA in liver cancer cell

**DOI:** 10.1038/srep18100

**Published:** 2015-12-11

**Authors:** Feng Yu, Xingyong Shen, Li Fan, Zhaocai Yu

**Affiliations:** 1 Department of Medical Oncology, The People’s Hospital of Shaanxi Province, Xi’an, P.R. China; 2 Department of Medical Oncology, Xijing Hospital, The Fourth Military Medical University, Xi’an, P.R. China

## Abstract

Human liver cancer is the cancer commonly seen clinically. The transcription of ribosomal DNA (rDNA) is a critical step for cells, and epigenetic marks such as post-translational histone modifications have been involved in the regulation of rDNA transcription. But less is known about the pathogenesis of the liver cancers concerning the rDNA transcription regulation. Here we aligned the ChIP-seq data of histone modification markers and CTCF to the human genome assembly which contains a single rDNA repeat in human liver cancer cell and validated their distribution with ChIP-QPCR. Human liver cancer cell possesses a higher enrichment of H3K4me1 and H3K27me3 at ~28 kb within the intergenic spacer (IGS) of rDNA and a higher enrichment of H3K4me3 and H3K27ac upstream of TSS. Furtherly, we studied whether UBF could affect histone modification markers and CTCF at rDNA in human liver cancer cell. UBF depletion leads to a decrease of gene activation mark H3K4me3 across the rDNA promoter. And other histone modification marks and CTCF were not altered after UBF depletion. Taken together, our data showed a high resolution map of histone modification marks at rDNA in human liver cancer cell and provide novel evidence to decipher chromatin-mediated regulation of rDNA in liver cancer.

Hepatocellular carcinoma is one of the most frequent cancers in the world. The transcription of human ribosomal DNA (rDNA) plays a vital role for life, which can represent almost 80% of all the cellular RNA production^1^. Dysregulation of rDNA transcription has been implicated in cancers^2^. In human, there are ~300–400 copies of rDNA gene on each haploid genome arrayed tandemly in nucleolar organizer regions (NORs) on the five chromosomes 13, 14, 15, 21 and 22^3^,^4^. Each unit of a 43kb rDNA repeat contains ~13.3 kb coding region and an ~30 kb intergenic spacer (IGS) containing the enhancer and the promoter of the rDNA gene^3^.

The transcription of rDNA by RNA Polymerase I generates a 47S pre-rRNA precursor which can be then processed and produce the mature ribosomal RNA including 18S, 5.8S and 28S units. Epigenetic modifications have been involved in the regulation of human ribosomal DNA transcription^5^,^6^,^7^,^8^,^9^. Only a fraction of the rDNA genes in eukaryotic cells is active, while others are silenced. The transcriptionally active and repressed rDNA genes can be characterized by different epigenetic marks including histone post-translational modifications, the methylation of ribosomal DNA, etc. The transcriptionally inactive rDNA is associated with heterochromatin, hypermethylated at CpG sites and characterized with repressive histone modification markers such as H3K27me3. Transcriptionally active rDNA is generally associated with euchromatin, hypomethylated and marked with histone modifications usually associated with gene activation such as H3K4me3^3^,^10^.

Previous reports showed that transcription termination factor-1 (TTF-1) recruited to the rDNA T0 site can bring the nucleolar remodeling complex (NoRC) to the rDNA promoter. NoRC is one factor of ATP-dependent chromatin remodeling machines and is composed of two subunits: the TIP5 and ATPase SNF2H. NoRC could silence rDNA transcription through recruiting enzymes modifying chromatin including DNA methyltransferase (DNMT), histone methyltransferase (HMT), histone deacetylases, i.e. HDAC1 and through shifting the nucleosome bound to promoter into a silent position^5^,^6^,^11^,^12^,^13^,^14^,^15^, and it could also mediate the formation of a closed nucleosomal structure^7^. Another mechanism exists for activating rRNA genes: TTF1 can recruit Cockayne Syndrome Group B protein (CSB) to the active ribosomal DNA promoter thus activate rDNA transcription^13^. The mechanisms which decide the recruitment of CSB versus NoRC and thus are responsible for the state of rDNA transcription remain an open question to be explored.

Particularly, DNA methylation mediated by NoRC has been involved in the repression of rDNA transcription^16^ and has also been shown to decrease Upstream Binding Factor (UBF) binding to the rDNA promoter^17^. UBF, which is involved in the formation of the PolI preinitiation complex (PIC)^1^,^17^,^18^, has been shown to be involved in the regulation of rDNA transcription^17^. UBF has previously been shown to decide the number of active ribosomal RNA genes, and knockdown of UBF results in a modest decrease of rDNA transcription in murine cells^17^.

Increasing evidences suggest a regulational machinery exists to prevent the inappropriate transcription as human ribosomal DNA is organized into Nucleolar Organizing Regions (NORs) which are tandemly repeated. Such a mechanism could be involving insulator elements, the discrete transcriptional units are generally demarcated and the leaky transcription was prevented by insulator elements^19^.

One of the insulator-binding proteins that are well-characterized is CTCF^20^. Previous reports showed that CTCF is localized in the nucleolus and is required for the repression of human ribosomal DNA transcription^21^.

The rapidly-increasing development of ChIP-seq^22^ has made it possible to detect the protein occupancy throughout the human genome. In this report, we first showed the distribution of histone modification markers at rDNA in human liver cancer cell HepG2 by analyzing the ChIP-seq datasets and validated the ChIP-seq results by ChIP-QPCR. The locations of the five histone modifications at human rDNA were presented here, expanding the previous study of histone modification distribution at rDNA in some other cell lines to the human liver cancer cell. Secondly, we studied the effect of UBF on the distributions of these repressive and active histone modification marks at rDNA in HepG2 cell line. We also report the presence of CTCF, an insulator-binding protein, at the rDNA space promoter in human liver cancer cell.

Altogether, our studies showed for the first time a high-resolution map of histone modification marks and CTCF at rDNA in human liver cancer cell, and give evidence for future research of chromatin-mediated regulation of rDNA in liver cancer.

## Results

### Location of histone modifications at human ribosomal DNA in liver cancer cell

Around 300–400 copies of rDNA arrange on several chromosomes in tandemly repeated arrays in the human genomes^1^,^3^. However, human rDNA sequence is not contained in the human genome assemblies because the rDNA sequencing was not performed during the human genome sequencing^16^,^17^. Therefore, we added the consensus sequence of one copy of rDNA repeat to the current human genome assembly HG19 as described previously^23^. In brief, we added the rDNA sequence to the chromosome 13 at the proximal tip, which generated the assembly called HG19_plus_rDNA. Reads which were aligned to more than one region in the human genome were discarded to reduce false positive signals.

The publically available (the ENCODE consortium) ChIP-seq data for human liver cancer cell (HepG2) were aligned to the assembly HG19_plus_rDNA and the distribution of different histone post-translational modifications at ribosomal DNA was analyzed.

Totally five histone modifications were analyzed: two promoter and enhancer histone modification marks (H3K4me1 and H3K27ac), one transcriptional activation marker (H3K4me3), one usually associated with the transcription elongation (H3K36me3) and the last one generally associated with the transcription repression (H3K27me3).

**Figure 1:**
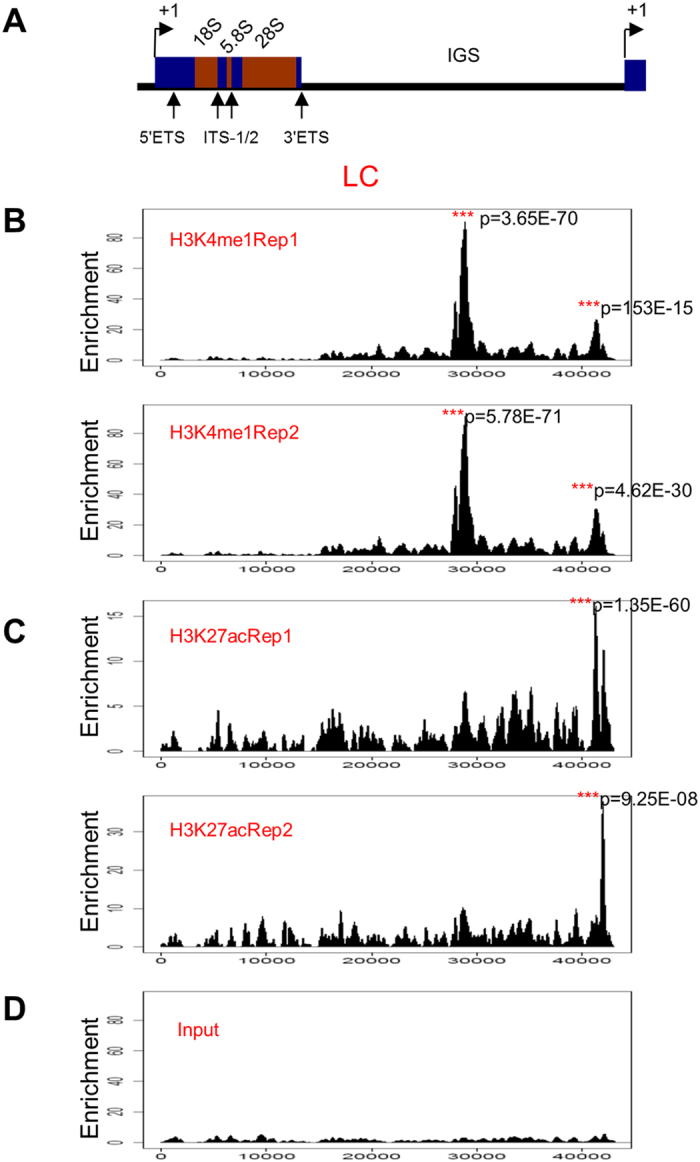
Distribution of histone modification marks for the promoter and enhancer elements at rDNA in human liver cancer cell. (**A**) Schematic representation of one human rDNA repeat. ETS: external transcribed spacer; ITS-1/2: internal transcribed spacer-1/2; IGS: intergenic spacer. The coding region, which spans ~0–13.3 kb of the human rDNA repeat, is separated by the non-coding intergenic spacers (IGS). (**B**) Patterns of histone modifications H3K4me1 at rDNA in human liver cancer cell HepG2 were shown below the human rDNA repeat as determined by the analysis of ChIP-seq data. Enrichment P value was calculated using the negative binomial model. (**C**) Patterns of histone modifications H3K27ac at rDNA in human liver cancer cell HepG2 were shown below the human rDNA repeat as determined by the analysis of ChIP-seq data. Enrichment P value was calculated using the negative binomial model. (**D**) Pattern of human liver cancer cell HepG2 Input at rDNA was shown below the human rDNA repeat as determined by the analysis of ChIP-seq data.

**Figure 2:**
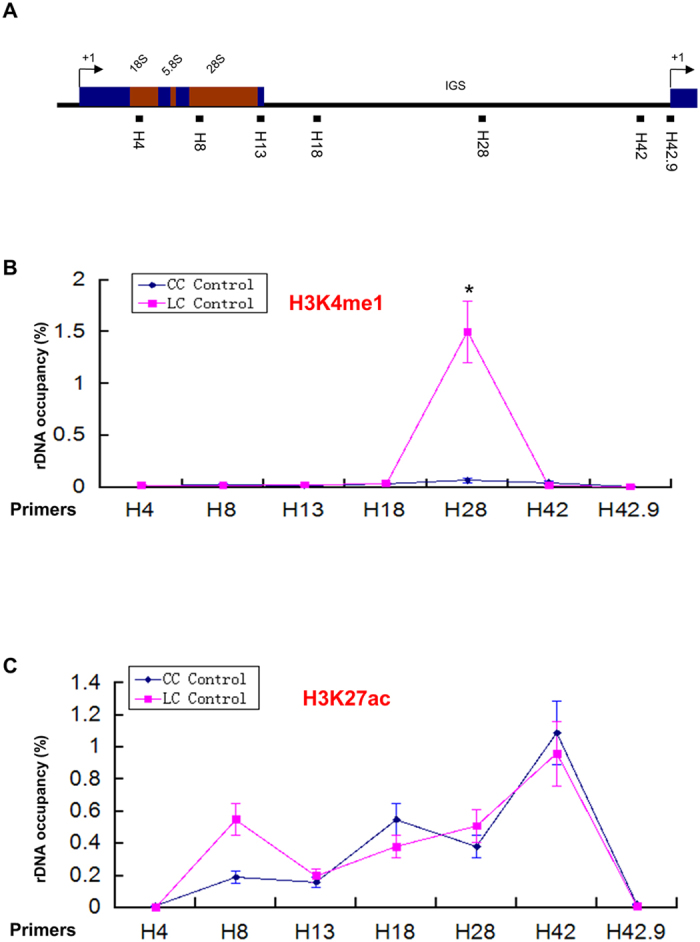
ChIP-QPCR for histone modifications (H3K4me1 and H3K27ac) in human liver cancer cell (LC control) and normal liver cell (CC control). (**A**) Schematic representation of one human rDNA repeat. The positions of QPCR amplicons in ChIP assays are indicated with solid bars. (**B**) Enrichment of H3K4me1 on rDNA analyzed by ChIP-QPCR. Chromatin from HepG2 cells was cross-linked and immunoprecipitated with H3K4me1 antibody, DNA was analyzed by QPCR with different sets of primers indicated in (**A**). The percentage of DNA associated with anti-H3K4me1 antibody was calculated relative to the DNA from ChIP input. Values are represented by means ± SD derived from three independent ChIP experiments, each experiment was tested by at least three independent QPCR reactions. Student’s t-test was performed between human liver cancer cell (LC Control) and normal liver cell (CC Control). (**C**) Enrichment of H3K27ac on rDNA analyzed by ChIP-QPCR. Chromatin from HepG2 cells was cross-linked and immunoprecipitated with H3K27ac antibody, DNA was analyzed by QPCR with different sets of primers indicated in (**A**). The percentage of DNA associated with anti-H3K27ac antibody was calculated relative to the DNA from ChIP input. Values are represented by means ± SD from three independent ChIP experiments, each experiment was tested by at least three independent QPCR reactions. Student’s t-test was performed between human liver cancer cell (LC Control) and normal liver cell (CC Control).

We found the H3K4me1 enrichment near the rDNA promoter region and in a site which is located ~28 kb within the IGS of human rDNA repeat in the human liver cancer cell (Fig. 1B), which is consistent with the previous analysis of rDNA distribution for histone modification marker H3K4me1^23^. ChIP-QPCR (Fig. 2B, Primer H28) confirmed the higher enrichment of H3K4me1 at IGS region in liver cancer cell line HepG2 compared to that of normal liver cells. However, no significant difference was found for the region near the rDNA promoter (Fig. 2B, Primer H42). Although the significance of the H3K4me1 occupancy at IGS region of human rDNA is currently unknown, the result indicates that the functional role of H3K4me1 may differ between cancer cells and normal cells in the human liver.

The ChIP-seq data of H3K27ac, another histone modification marker of promoter and enhancer region, shows that human liver cancer cell HepG2 possesses enrichment in the region just upstream of the TSS (Fig. 1C), which was verified by ChIP-QPCR (Fig. 2C).

**Figure 3:**
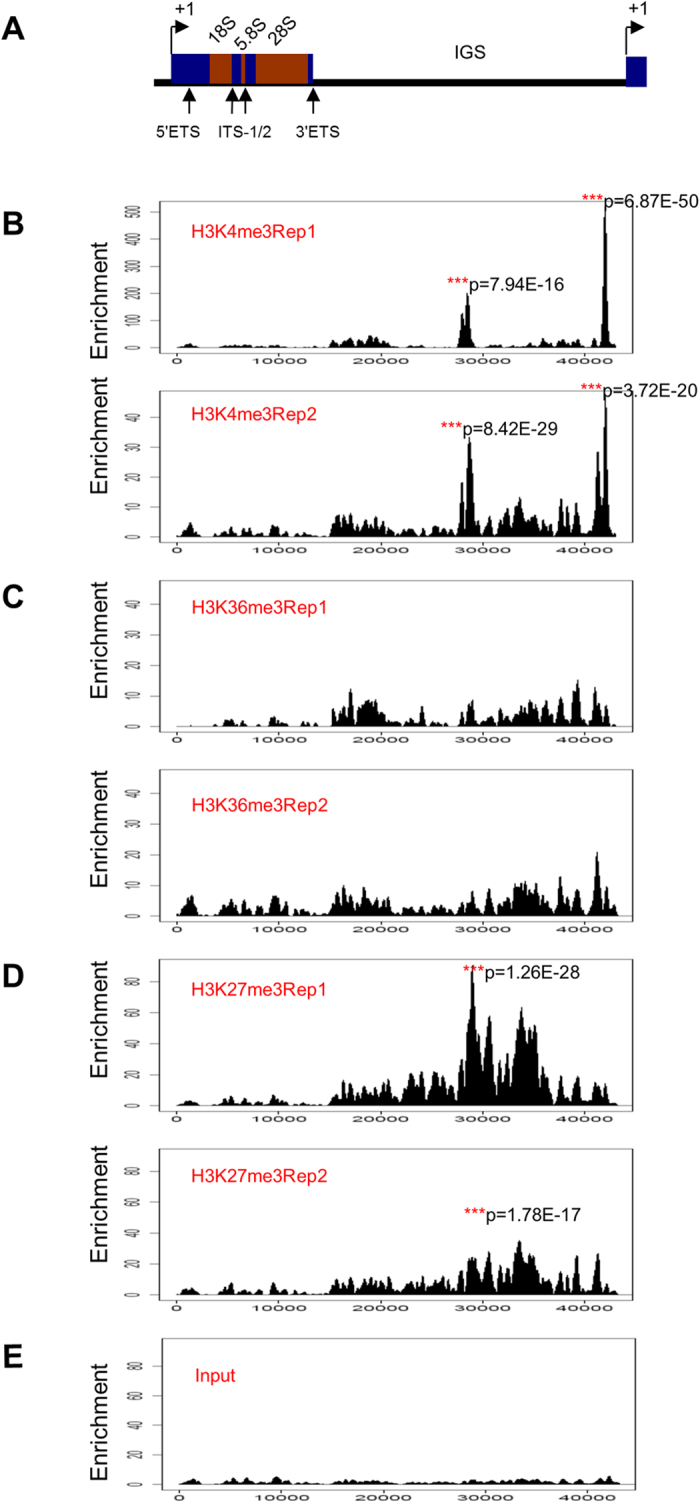
Distribution of histone modification markers (H3K4me3, H3K36me3 and H3K27ac) at rDNA in human liver cancer. (**A**) Schematic representation of one human rDNA repeat. ETS: external transcribed spacer; ITS-1/2: internal transcribed spacer-1/2; IGS: intergenic spacer. The coding region, which spans ~0–13.3 kb of the human rDNA repeat, is separated by the non-coding intergenic spacers (IGS). (**B**) Patterns of histone modifications H3K4me3 at rDNA in human liver cancer cell HepG2 were shown below the human rDNA repeat as determined by the analysis of ChIP-seq data. Enrichment P value was calculated using the negative binomial model. (**C**) Patterns of histone modifications H3K36me3 at rDNA in human liver cancer cell HepG2 were shown below the human rDNA repeat as determined by the analysis of ChIP-seq data. Enrichment P value was calculated using the negative binomial model. (**D**) Patterns of histone modifications H3K27me3 at rDNA in human liver cancer cell HepG2 were shown below the human rDNA repeat as determined by the analysis of ChIP-seq data. Enrichment P value was calculated using the negative binomial model. (**E**) Pattern of human liver cancer cell HepG2 at rDNA was shown below the human rDNA repeat as determined by the analysis of ChIP-seq data.

**Figure 4:**
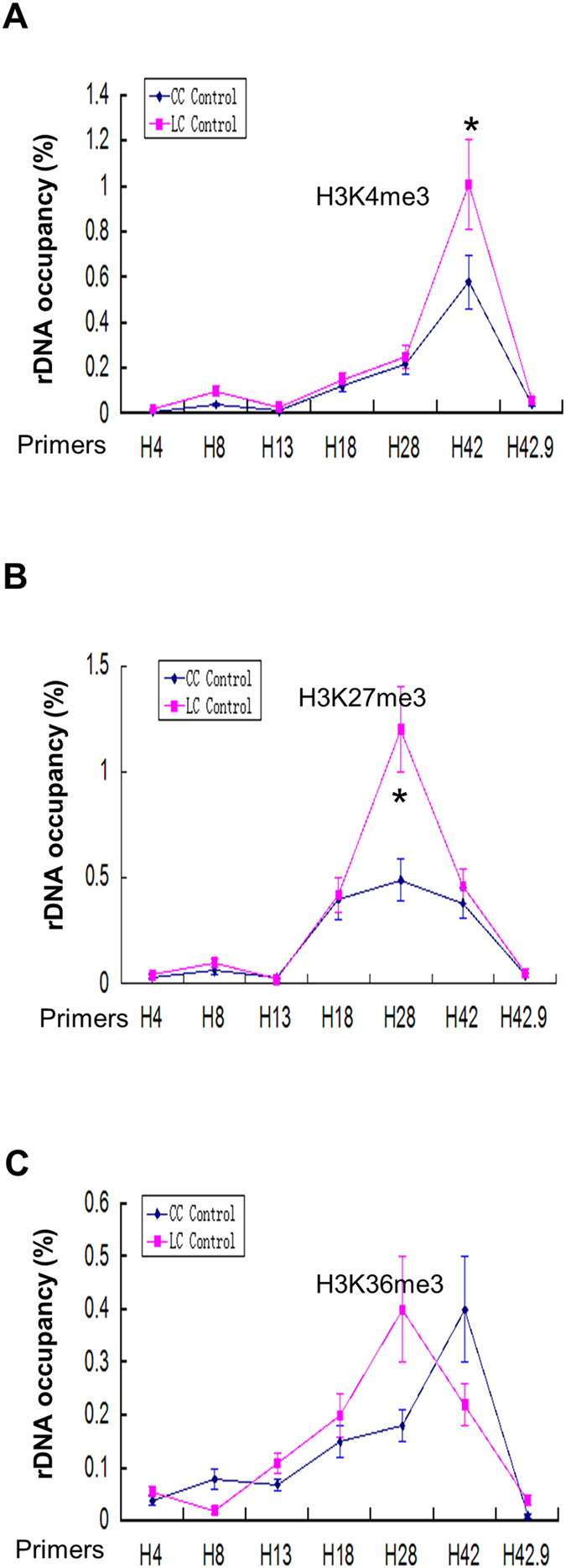
ChIP-QPCR for histone modifications (H3K4me3, H3K36me3 and H3K27ac) in human liver cancer cell (LC control) and normal liver cell (CC control). (**A**) Enrichment of H3K4me3 on rDNA analyzed by ChIP-QPCR. Chromatin from HepG2 cells was cross-linked and immunoprecipitated with H3K4me3 antibody, DNA was analyzed by QPCR with different sets of primers indicated in (**A**). The percentage of DNA associated with anti-H3K4me3 antibody was calculated relative to the DNA from ChIP input. Values are represented by means ± SD from three independent ChIP experiments, each experiment was tested by at least three independent QPCR reactions. (**B**) Enrichment of H3K27me3 on rDNA analyzed by ChIP-QPCR. Chromatin from HepG2 cells was cross-linked and immunoprecipitated with H3K27me3 antibody, DNA was analyzed by QPCR with different sets of primers indicated in (**A**). The percentage of DNA associated with anti-H3K27me3 antibody was calculated relative to the DNA from ChIP input. Values are represented by means ± SD from three independent ChIP experiments, each experiment was tested by at least three independent QPCR reactions. Student’s t-test was performed between human liver cancer cell (LC Control) and normal liver cell (CC Control). (**C**) Enrichment of H3K36me3 on rDNA analyzed by ChIP-QPCR. Chromatin from HepG2 cells was cross-linked and immunoprecipitated with H3K27me3 antibody, DNA was analyzed by QPCR with different sets of primers indicated in (**A**). The percentage of DNA associated with anti-H3K36me3 antibody was calculated relative to the DNA from ChIP input. Values are represented by means ± SD derived from three independent ChIP experiments, each experiment was tested by at least three independent QPCR reactions. Student’s t-test was performed between human liver cancer cell (LC Control) and normal liver cell (CC Control).

The regions enriched by H3K4me1 in the human liver cells also show enrichment of H3K4me3, a histone modification marker for transcriptional activation, in liver cancer cell (Fig. 3B), H3K4me3 ChIP-QPCR was used to confirm enrichment at these two regions (Fig. 4A). Intriguingly, human liver cancer cell possesses a higher enrichment near the TSS region (H42) than in normal liver cell (Fig. 4A). Little enrichment of histone modification markers for gene activation was found in the coding region of human rDNA repeat in HepG2 cells, which is in support of the previous speculation that the coding region of active human ribosomal DNA copies might be poor of nucleosomes^19^,^24^,^25^,^26^.

Generally, distribution of repressive histone modification markers which are distributed broadly along the IGS such as H3K27me3 (Fig. 3D) is less punctuate than active modifications.

Intriguingly, H3K36me3, a histone modification mark associated with transcription elongation, is present along the whole human rDNA repeat, from the coding region to the IGS region (Fig. 3C). The ChIP-QPCR of H3K27me3 and H3K36me3 showed a relatively similar enrichment of histone modifications of rDNA as compared with that of ChIP-seq results (Fig. 4B,C). And a higher enrichment of H3K27me3 at the IGS region of human rDNA was also discovered in human liver cancer cell than in normal liver cell.

### UBF depletion does not alter DNA methylation and histone modification markers for gene repression

**Figure 5:**
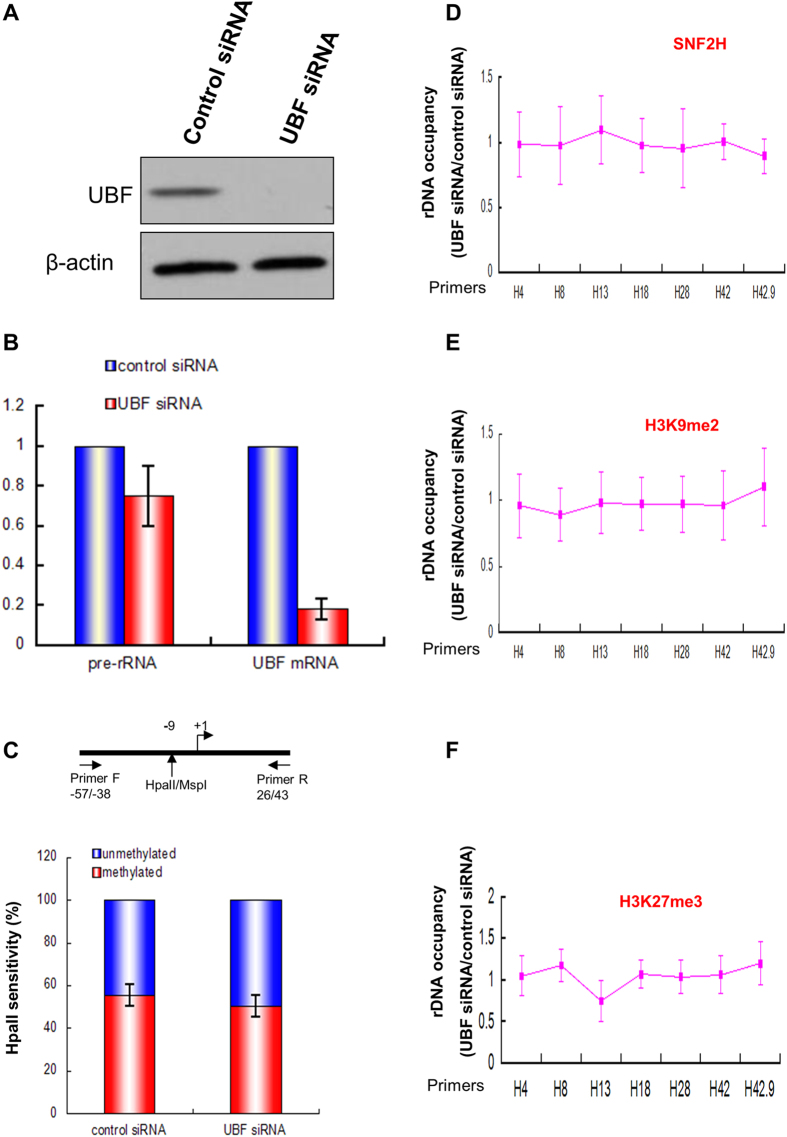
UBF depletion does not affect DNA methylation and other heterochromatic markers. (**A**) Knockdown of UBF expression by siRNA. Human liver cancer cell (HepG2) was transfected with control or UBF siRNA, and protein samples were harvested and analyzed by Western blot (UBF and β-actin) 48 h after transfection. The gels have been run under the same experimental conditions. The full-length blot is presented in Supplementary Figure 2A. (**B**) Levels of UBF mRNA and pre-rRNA were determined by RT-QPCR. The data are derived from three different independent experiments. **p < 0.01, *0.01 < p < 0.05, Student’s t-test was done for cells transfected with or without UBF siRNA. (**C**) Depletion of UBF does not affect the methylation status of rDNA promoter. DNA was isolated from HepG2 cells transfected with control siRNA or UBF siRNA and digested with MspI or HpaII. The promoter region of human rDNA was then amplified with the primers flanking the CCGG site at −9. Only the methylated CpG dimer was resistant to cleavage with HpaII, it can yield a fragment of 100 nt that is amplified from −57 to +43 of rDNA, whereas unmethylated DNA would be cleaved and no PCR product would be amplified. Student’s t-test was performed between cells transfected with or without UBF siRNA. (**D–F**) Depletion of UBF does not alter the binding of SNF2H or histone modifications associated with the silencing of rDNA mediated by NoRC. ChIP-QPCR analysis of different regions of rDNA in HepG2 cells transfected with control or UBF siRNA using antibodies against SNF2H (**D**), H3K9m2 (**E**), H3K27me3 (**F**). The ratio of rDNA occupancy between UBF knockdown cells and control cells was calculated for each antibody. Student’s t-test was performed between cells transfected with or without UBF siRNA.

To test whether UBF plays a role in the regulation of ribosomal DNA transcription in human liver cancer cell, UBF was knocked down by siRNA transfection (Fig. 5A) and we quantified the pre-rRNA level (Fig. 5B). The level of 5′ external transcribed spacer (ETS) was reduced about 20% in response to UBF depletion (Fig. 5B).

To determine whether the decreased level of rDNA transcription was associated with an increased silencing mediated by the methylation of ribosomal DNA, the methylation level of the promoter of ribosomal DNA was detected in HepG2 cells transfected with or without UBF siRNA. UBF knockdown did not lead to an alteration of ribosomal DNA methylation level at this promoter site (Fig. 5C). Moreover, UBF depletion did not lead to a change of the enrichment of SNF2H, a component of the NoRC complex^6^,^27^,^28^, at rDNA promoter and coding region (Fig. 5D). In addition, the histone modification marks associated with NoRC-dependent gene repression such as H3K9me2, H3K27me3 remain unaltered after UBF knockdown (Fig. 5E,F). Therefore, the UBF inactivation can induce a modest decrease of pre-rRNA level without increasing the methylation of rDNA promoter and other NoRC-mediated chromatin remodeling machineries.

### Depletion of UBF results in a decrease of H3K4me3 upstream of TSS of rDNA

**Figure 6:**
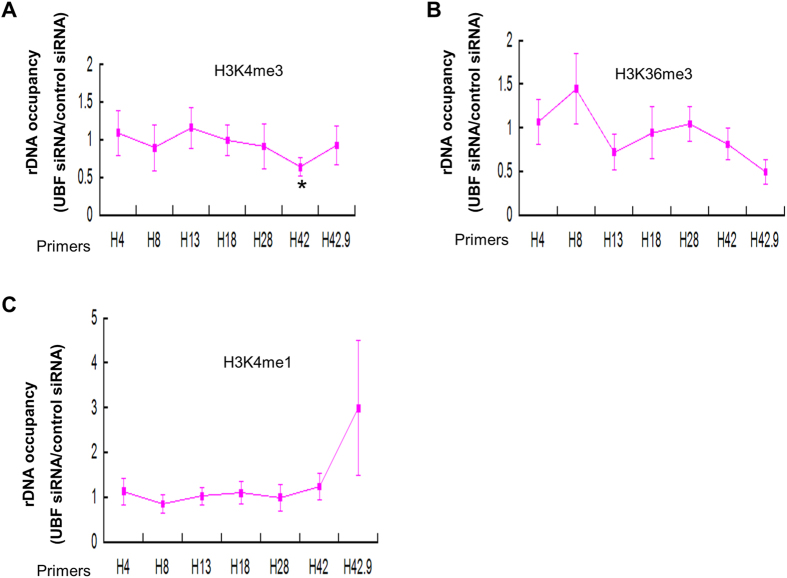
Effect of UBF depletion on the distribution of histone modification marks (H3K4me3, H3K36me3, H3K27me3 and H3K4me1) at rDNA in human liver cancer cell. (**A**) Loss of UBF leads to a decrease of H3K4me3 at H42 region of rDNA. ChIP-QPCR analysis of different regions of rDNA in HepG2 cells transfected with control or UBF siRNA using antibodies against H3K4me3. The ratio of rDNA occupancy between UBF knockdown cells and control cells was calculated for each antibody. Student’s t-test was performed between cells transfected with or without UBF siRNA. (**B–C**) Depletion of UBF does not alter H3K36me3 and H3K4me1 distribution at rDNA. ChIP-QPCR analysis of different regions of rDNA in HepG2 cells transfected with control or UBF siRNA using antibodies against H3K36me3 (**B**), H3K4me1 (**C**). The ratio of rDNA occupancy between UBF knockdown cells and control cells was calculated for each antibody. Student’s t-test was performed between cells transfected with or without UBF siRNA.

Intriguingly, we found that knockdown of UBF depletion leads to an increase of euchromatic marks H3K4me3 across the ribosomal DNA promoter in human liver cancer cell (Fig. 6A), This is not due to the increased level of H3K4me3 as UBF knockdown does not result in any significant change of H3K4me3 level in HepG2 cell line (Supplementary Figure S1). No difference was discovered for the histone modification markers H3K36me3 and H3K4me1 at rDNA after the knockdown of UBF in human liver cancer cell (Fig. 6B,C).

### CTCF is associated with human ribosomal DNA in liver cancer cell

**Figure 7:**
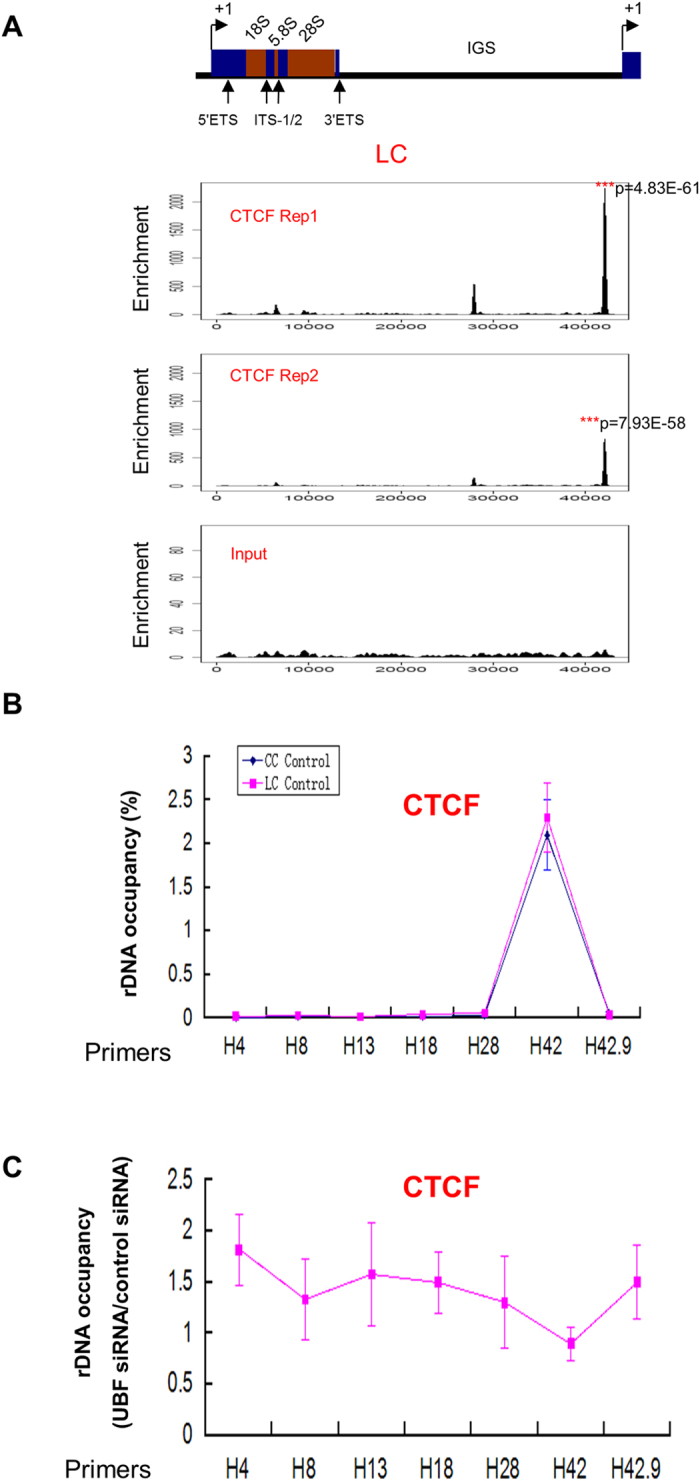
Distribution of CTCF at rDNA in human liver cancer cell. (**A**) Schematic representation of one human rDNA repeat. ETS: external transcribed spacer; ITS-1/2: internal transcribed spacer-1/2; IGS: intergenic spacer. The coding region, which spans ~0–13.3 kb of the human rDNA repeat, is separated by the non-coding intergenic spacers (IGS). Patterns of CTCF at rDNA in human liver cancer cell line HepG2 were shown below the human rDNA repeat as determined by the analysis of ChIP-seq data. Enrichment P value was calculated using the negative binomial model. (**B**) Enrichment of CTCF on human rDNA analyzed by ChIP-QPCR. Chromatin from HepG2 cells was cross-linked and immunoprecipitated with CTCF antibody, DNA was analyzed by QPCR with different sets of primers indicated in (**A**). The percentage of DNA associated with anti-CTCF antibody was calculated relative to the DNA from ChIP input. Values are represented by means ± SD from three independent ChIP experiments, each experiment was tested by at least three independent QPCR reactions. Student’s t-test was performed between human liver cancer cell (LC Control) and normal liver cell (CC Control). (**C**)Depletion of UBF leads to no change of CTCF at human rDNA. ChIP-QPCR analysis of different regions of rDNA in HepG2 cells transfected with control or UBF siRNA using antibodies against CTCF. The ratio of rDNA occupancy between UBF knockdown cells and control cells was calculated for each antibody. Student’s t-test was performed between cells transfected with or without UBF siRNA.

We aligned ChIP-seq data of CTCF from human liver cancer cell HepG2 to HG19_plus_rDNA and we have discovered that in human hepatic cancer cells, CTCF was highly enriched at the 3′ end of human ribosomal DNA just upstream of TSS (Fig. 7A,B).

Next we tested whether UBF can affect the distribution of CTCF on rDNA, and no change was found in the distribution of CTCF on rDNA after UBF depletion in human liver cancer cell HepG2 (Fig. 7C).

## Discussion

Primary liver cancer is the sixth most frequent cancer worldwide and the second leading cause of cancer death, thus it is important to study the mechanism of liver cancerology for the clinical therapy. As previous studies have suggested that rDNA transcription by RNA Polymerase I is tightly linked to cancerology^9^ and epigenetic mechanisms are involved in the control of transcriptional activity of rRNA genes, in particular, histone modifications are a key issue to mediate the regulation of rDNA transcription. Here we first showed the distribution of histone modification at rDNA locus in human liver cancer cell. We found that in human liver cancer cell, histone modifications usually associated with gene activation at rDNA are distributed more punctately than those repressive histone modification markers whose distribution is much broader at rDNA. Importantly, we found a high peak of H3K4me1 at the ~28–29 kb site within the IGS of rDNA in human liver cancer cell, while this peak does not exist in the normal human liver cells. The significance of the H3K4me1 peak in the human liver cancer cell at the ~28–29 kb site within IGS of human rDNA is still unclear. It has been suggested before that this region may possess an unknown transcription unit or a novel functional element such as an enhancer^23^ consistent with the notion that H3K4me1 is a histone modification mark for enhancers. The absence of this H3K4me1 peak in the human normal liver cells may suggest a potential mechanism of the pathogenesis of liver cancer. However, the limitation of the ChIP-seq analysis in this paper is that the aligned signal obtained at human ribosomal DNA repeat is an aggregate of signal for all human ribosomal DNA repeats and does not discriminate the transcriptionally active and silenced ribosomal DNA, therefore, the ChIP-seq analysis data show an average for both silenced and active populations. Further study using reporter assays with this region of human ribosomal DNA will be done to address this issue.

The association of TTF-1 to T0 allows the recruitment of the nucleolar remodeling complex NoRC to T0^3^,^7^,^9^, NoRC can then recruit histone deacetylase and histone methyltransferase to generate a structure of heterochromatin rDNA to repress rDNA transcription^5^,^6^,^7^. UBF plays a vital role in the rDNA transcription^1^. Our data showed that loss of UBF leads to about 20% decrease of rDNA transcription in human liver cancer cell line HepG2 cells. UBF knockdown did not affect the methylation status of rDNA and other heterochromatic markers (Fig. 5) demonstrating that NoRC chromatin remodeling machinery is not required for the rDNA silencing after UBF depletion in HepG2 cells, which is consistent with previous report for murine cells^17^. Of note, silencing of the rDNA transcription independent of DNA methylation was also observed after the inactivation of nucleolin or macroH2A1^29^,^30^. However, previous psoralen analysis of the rRNA genes showed a 70% decrease in the fraction of active genes after UBF depletion in murine NIH3T3 cells, and this psoralen cross-linking analysis needs to be confirmed in human liver cancer cell. In addition, UBF depletion results in a decrease of H3K4me3 in the upstream of TSS in human liver cancer cell (Fig. 6A, H42) suggesting H3K4me3 is involved in the UBF-mediated rDNA transcription regulation in liver cancer.

CTCF, an insulator-binding protein, may mediate rDNA transcription either positively or negatively in a context-dependent manner^21^,^31^. In our study, CTCF was shown to be highly enriched at the 3′ end of human ribosomal DNA in human liver cancer cell, consistent with the previous report^23^. This may suggest that the 3′ of rDNA may play a role of an insulator element to discriminate the boundaries of the repeated human ribosomal DNA units. However, UBF depletion did not lead to an alteration of CTCF distribution at rDNA, suggesting that UBF is not involved in CTCF-mediated rDNA transcription regulation.

Taken together, our study compared the distribution of histone modification markers and CTCF at rDNA in human liver cancer cell with that in normal human liver cells.

Our data provide a novel insight into the UBF-mediated epigenetic mechanisms in the rDNA transcription in liver cancer and may be served as a reference for further studies of chromatin-mediated regulation of rDNA in liver cancer.

## Materials and Methods

### Cell culture and siRNA transfection

Human hepatocellular carcinoma cell line HepG2 were cultured in Dulbecco’s modified Eagle medium (DMEM, Gibco/Life Technologies) supplemented with 10% foetal bovine serum (FBS) (Gibco/Life Technologies) and 1% penicillin-streptomycin at 37 °C, 5% CO_2_. For the depletion of UBF, HepG2 cells were transfected with Lipofectamine 2000 (Life Technologies) using UBF siRNA or control siRNA (Life Technologies) as described previously^23^. The siRNA sequences against UBF are: AUCUCACUCAGCUCUCUCUCAUAUC. Cells were harvested 48 h after transfection for analysis, proteins were analyzed by Western Blot and RNAs were analyzed by reverse transcriptase-polymerase chain reaction (RT-QPCR).

### Transcription analysis by RT-QPCR

The total RNA from the cells was extracted by TRIzol (Life Technologies) according to standard methods. 100 ng of the total RNAs were reverse-transcribed with random primers and 1^st^-strand cDNA was prepared using First Strand cDNA synthesis Kit (Fermentas), and the synthesized cDNA was used as template for QPCR with EXPRESS One-Step SYBR GreenER SuperMix Kits (Life Technologies). The sequences of the used primers are listed in Table 1.

### Chromatin Immunoprecipitation (ChIP)

Chromatin Immunoprecipitation (ChIP) assays were performed as described earlier^32^,^33^. Cross-linking of HepG2 cells was done for 10 min in the cell culture medium with 1% formaldehyde at room temperature. 10^6^ HepG2 cells were used for each ChIP. The HepG2 cells were dissolved in 200 μl of sodium dedecyl sulphate (SDS) lysis buffer (10 mM EDTA, 50 mM Tris, pH 8.1, 1% SDS), and the sonication was performed to generate DNA fragments of 200–1000 bp. 10-fold dilution of chromatin was performed with ChIP dilution buffer (1.2 mM EDTA, 1.1% Triton X-100, 16.7 mM Tris, pH 8.1, 167 mM NaCl, 0.01% SDS). Chromatin was precipitated with their respective antibodies. Immunoprecipitated complexes of protein and DNA were reverse cross-linked and extracted with phenol/chloroform and precipitated with ethanol to get the purified DNAs.

The purified DNAs were amplified by QPCR using QuantStudio 6 Flex Real-Time PCR System (Life Technologies) and EXPRESS One-Step SYBR GreenER SuperMix Kits (Life Technologies). Primers for QPCR were reported^2^ or designed and listed in Table 1.

### Data sets

Data for ChIP-seq of histone modification markers, CTCF and their corresponding input from HepG2 cell line were retrieved from SRA: SRX099898 for H3K4me1, SRX067494 for H3K27ac, SRX067488 for H3K4me3, SRX067436 for H3K36me3, SRX067505 for H3K27me3, SRX067536 for CTCF, SRX067483 for Input.

### Alignment and analysis of ChIP-seq data

As human ribosomal DNA is not assembled in the human genome yet, a newly-built assembly of HG19 was created as described previously^23^. The bases of the unsequenced centromere region of chromosome 13 were removed, and a full, human ribosomal DNA unit (GenBank accession No. U13369) was added, which yields ‘rDNA_chr13’. A custom human genome build HG19 containing rDNA_chr13 instead of chromosome 13 was generated with the tool of bowtie-build^34^. This new HG19 assembly was designated ‘HG19_plus_rDNA’.

ChIP-seq data were aligned to “HG19_plus_rDNA” assembly with Bowtie tool^34^, permitting two mismatches per read. We discarded the reads with more than one reportable alignment during alignment with the ‘-m 1’ option. The ChIPseq peaks were called with the F-seq tool^35^ in which 200 was used for the fragment size for all analyses. The input samples were analyzed as described above and the signal was subtracted from the respective ChIP-seq data at each base with R.P value for the ChIP-Seq peak was calculated using the negative binomial model^36^,^37^.

### Antibodies

The following antibodies were used: anti-H3K4me1 (Abcam, ab8895); anti-H3K4me3 (Millipore, #07–473); anti-H3K27ac (Abcam, ab4729); anti-H3K36me3 (Cell Signaling, #9763S); anti-H3K27me3 (Millipore, #07–449); anti-CTCF (Millipore, #07–729); anti-β-actin (Sigma, A5441); anti-UBF (F-9) (Santa Cruz, sc-13125).

## Additional Information

**How to cite this article**: Yu, F. *et al.* Analysis of histone modifications at human ribosomal DNA in liver cancer cell. *Sci. Rep.* **5**, 18100; doi: 10.1038/srep18100 (2015).

## Supplementary Material

Supplementary Information

## Figures and Tables

**Table 1:** List of primer sequences.

**Primer name**	**5′ → 3′**	
	**Forward**	**Reverse**
human rDNA 5′-ETS	CCTGCTGTTCTCTCGCGCGTCCGAG	AACGCCTGACACGCACGGCACGGAG
human UBF	GTCGGCCATGTTCATCTTCT	CTCAGACAGGTCGTTCCACA
H4	CGACGACCCATTCGAACGTCT	CTCTCCGGAATCGAACCCTGA
H8	AGTCGGGTTGCTTGGGAATGC	CCCTTACGGTACTTGTTGACT
H13	ACCTGGCGCTAAACCATTCGT	GGACAAACCCTTGTGTCGAGG
H18	GTTGACGTACAGGGTGGACTG	GGAAGTTGTCTTCACGCCTGA
H28	TCCCCAGTTTTCAGGAAGAC	AACATCAACCGGCTCTCACT
H42	GGTTGTCGGGCTCCATCT	CTTTCCGGAGCTCTGCCTAG
H42.9	CCCGGGGGAGGTATATCTTT	CCAACCTCTCCGACGACA
GAPDH	TCCACCACCCTGTTGCTGTA	ACCACAGTCCATGCCATCAC
