# Supplementary material for: Analysis of histone modifications at human ribosomal DNA in liver cancer cell
Source: Sci Rep. 2015 Dec 11;5:18100. doi: 10.1038/srep18100 (PMC4676023; doi:10.1038/srep18100)
Supplement: Supplementary Information [file srep18100-s1.pdf]

# Analysis of histone modifications at human ribosomal DNA in liver cancer cell

**Feng Yu[1]§， Xingyong Shen[2]§, Li Fan[2], Zhaocai Yu[2]***

1. Department of Medical Oncology, The People's Hospital of Shaanxi Province, Xi'an, P.R. China
2. Department of Medical Oncology, Xijing Hospital, The Fourth Military Medical University, Xi'an, P.R. China

§ These authors contributed equally to this work

*Corresponding author: Zhaocai Yu, Department of Medical Oncology, Xijing Hospital, The Fourth Military Medical University, NO15, The West Changle Road, 710032, Xi'an, P.R. China; Email : yzhaocai@jinnchin.org

# SUPPLEMENTARY FIGURE LEGENDS

**Supplementary Figure S1. UBF depletion does not affect H3K4me3 level in HepG2 cell.**

(A) Human liver cancer cell (HepG2) were transfected with control or UBF siRNA, and protein samples were harvested and analyzed by Western Blot (UBF, H3K4me3, β-actin) 48h after transfection. The gels have been run under the same experimental conditions. The full-length blot is presented in Supplementary Figure 2B.

(B) Protein level in (A) was quantified with Image J. Student's t-test was done for cells transfected with or without UBF siRNA.

**Supplementary Figure S2. The full-length blots for the human liver cancer cell (HepG2) extracts transfected with control or UBF siRNA.**

(A) The full-length blot for Figure 5A. Human liver cancer cell (HepG2) were transfected with control or UBF siRNA, and protein samples were harvested and analyzed by Western Blot (UBF, β-actin) 48h after transfection.

(B) The full-length blot for Supplementary Figure 1A. Human liver cancer cell (HepG2) were transfected with control or UBF siRNA, and protein samples were harvested and analyzed by Western Blot (UBF, H3K4me3, β-actin) 48h after transfection.

**Supplementary Table S1. Effect of UBF depletion on H3K4me3 distribution at cancer-related genes in human liver cancer cell.**

ChIP-QPCR analysis of 15 cancer-related genes in HepG2 cells transfected with control or UBF siRNA using antibodies against H3K4me3. The ratio of H3K4me3 enrichment between UBF knockdown cells and control cells was calculated for each antibody. Student's t-test was performed between cells transfected with or without UBF siRNA. *: $0.01<p<0.05$.

**Supplementary Table S2. List of primer sequences used in ChIP-QPCR in Supplementary Table S1**

A

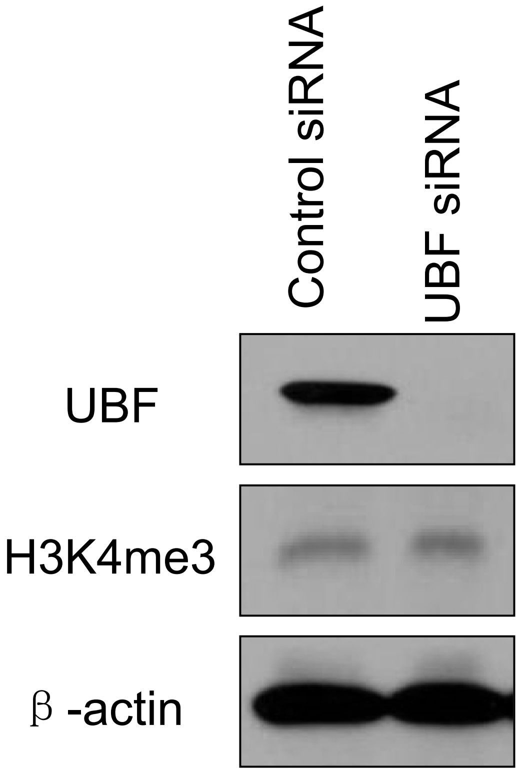


B

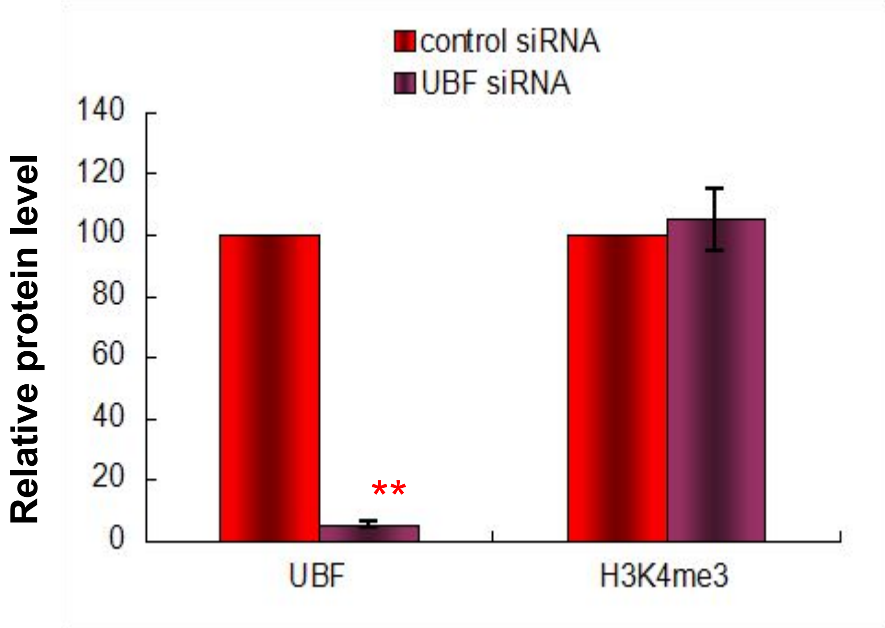


**Supplementary Figure S1. UBF depletion does not affect H3K4me3 level in HepG2 cell.**

**A**

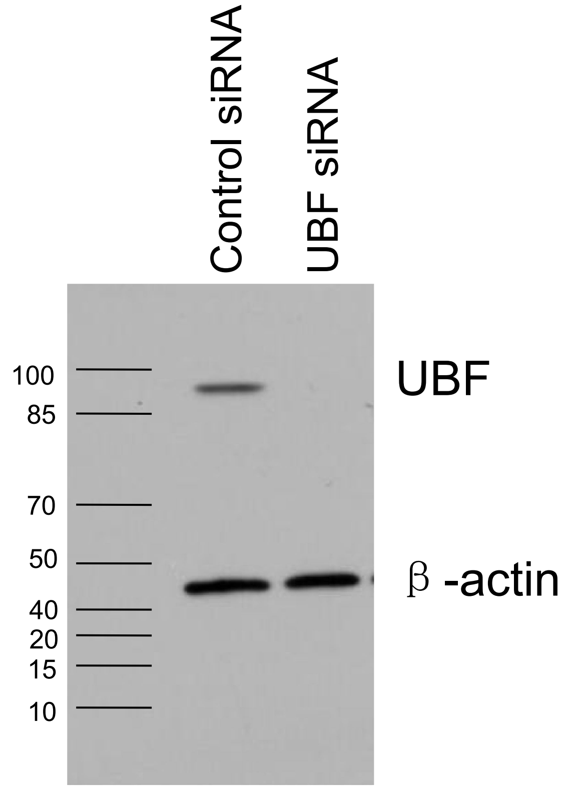


**B**

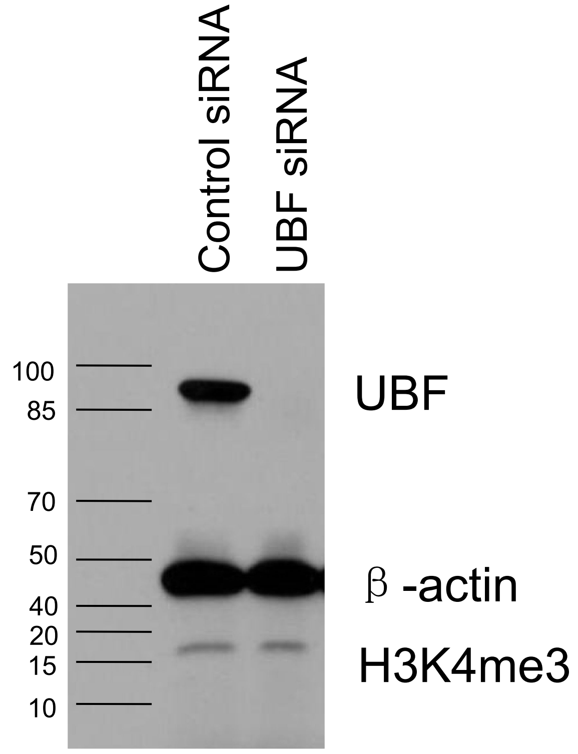


**Supplementary Figure S2. The full-length blots for the human liver cancer cell (HepG2) extracts transfected with control or UBF siRNA.**

**Supplementary Table S1. Effect of UBF depletion on H3K4me3 distribution at cancer-related genes in human liver cancer cell**

| **Gene** | **Ratio of H3K4me3 enrichment (UBF siRNA/control siRNA)** |
|---|---|
| CEA | 1.11±0.22 |
| CA19-9 | 1.05±0.18 |
| EGFR | 1.13±0.24 |
| ALK | 0.98±0.19 |
| AKT1 | 1.08±0.17 |
| AKT2 | 1.12±0.25 |
| AKT3 | 1.09±0.17 |
| PTEN | 1.10±0.15 |
| RB1 | 1.06±0.21 |
| ZNF668 | 0.99±0.18 |
| NPM | 1.56±0.09 * |
| KRAS | 1.02±0.12 |
| NOTCH1 | 1.04±0.14 |
| GNAS | 1.06±0.23 |
| GAPDH | 1.02±0.10 |

**Supplementary Table S2. List of primer sequences**

| Primer name | 5'→3' | |
|---|---|---|
| | **Forward** | **Reverse** |
| CEA | atctgaacctctcctgccac | tctgagttatgggcttggca |
| EGFR | ctgaaggaccctcggacttt | cttagagccagcgtcggata |
| ALK | ctcagatttgccagtgtcgc | gtgagaactggaagaggcct |
| AKT1 | ctccacgagttcctcctgtt | ggcgactcatgcagaaagag |
| AKT2 | ggctaccttgatgcttgctc | tgagcagagagatgtggcaa |
| AKT3 | agaatcgcttgaacctggga | gaaaaccagtcacgcctacc |
| PTEN | gagccggatgaggtgataca | ctctttccttttgcaccgct |
| RB1 | tatgtaaggtggccagcaca | agacacttgctggccttttg |
| ZNF668 | gctgcaagtagaacgtaggc | gggatcatggttgggaggaa |
| NPM | caccctccgagctctcttag | cagagagagtgaattgcggc |
| KRAS | cgttgaaagggtctgtcgtg | ggcgcgcatccatttactat |
| NOTCH1 | aacgagaagtagtcccaggc | gcactagtgaggctcagagt |
| GNAS | attccgtgtcaccctgaact | ccccacggttgaggtagtag |
| GAPDH | tccaccaccctgttgctgta | accacagtccatgccatcac |
